# Prescription and dispensing duration of medicines for hypertension and other chronic conditions: a review of international policies and evidence to inform the Australian setting

**DOI:** 10.1038/s41440-024-01743-2

**Published:** 2024-06-07

**Authors:** Peder af Geijerstam, Michael O. Falster, John Chalmers, Andrew J. McLachlan, Anthony Rodgers, Aletta E. Schutte

**Affiliations:** 1 Department of Health, Medicine and Caring Sciences, Faculty of Medicine and Health Sciences, Linköping University, Linköping, Sweden; 2 The George Institute for Global Health, University of New South Wales, Sydney, NSW Australia; 3 School of Population Health, University of New South Wales, Sydney, NSW Australia; 4 Sydney Pharmacy School, Faculty of Medicine and Health, University of Sydney, Sydney, NSW Australia

**Keywords:** Prescription duration, Dispensing duration, Medication adherence, Health outcomes, Health costs

## Abstract

The duration of treatment for which a physician may prescribe a medicine, ‘prescription duration’, is often dispensed at the pharmacy on multiple occasions of shorter time periods, ‘dispensing duration’. These durations vary significantly between and within countries. In Australia, the quantity of medication supplied at each dispensing has recently been extended from 30 to 60 days for a selection of medicines used for chronic health conditions, such as diabetes and hypertension. Dispensing durations vary between countries, with 30, 60 or 90 days being the most common—with 90 days aligning with the recommendation of the 2023 Global Report on Hypertension from the World Health Organization. The full impact of shorter vs longer prescription durations on health costs and outcomes is unknown, but current evidence suggests that 90-day dispensing could reduce costs and improve patient convenience and adherence. More rigorous research is needed.

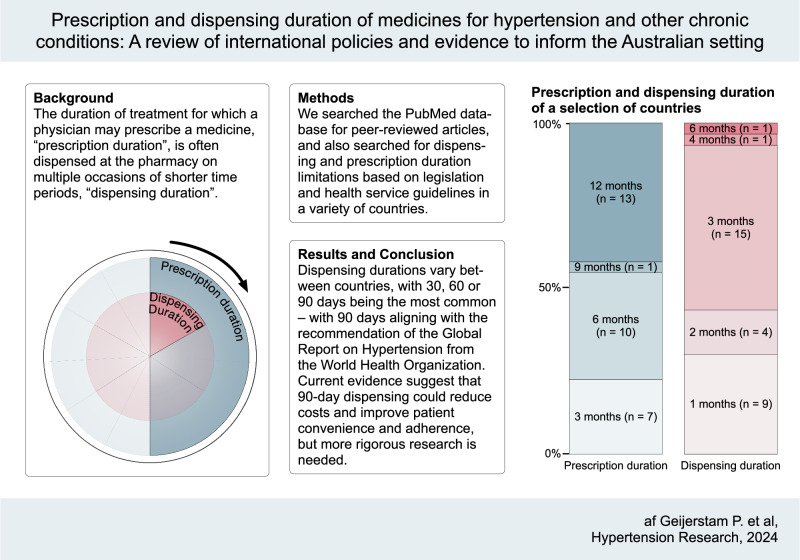

## Introduction

More than 11 million Australians have at least one chronic health condition, and 80.4% of those are dispensed a medication subsidised under the Australian Pharmaceutical Benefits Scheme (PBS) [1, 2]. Providing this care entails regular visits to a doctor for a prescription and a pharmacist for dispensing, which are mostly done in person. It may also involve substantial investments from patients in terms of out-of-pocket co-payments, travel costs and time commitments and from taxpayers, who fund the rest of the costs associated with doctor and pharmacist visits [3]. These costs can be substantial—for example a recent analysis showed that for the management of hypertension in primary care in 2022, patients and Australian taxpayers paid A$1.2 billion, and 42% came directly from out-of-pocket costs provided by consumers; of the A$1.2 billion, 51% was on pharmacy costs, 29% on general practitioner costs and 20% to manufacturers for the cost of medicines [4].

Australia recently introduced a policy extending the dispensing quantity supplied for a selection of medicines used for treating chronic conditions from 30 to 60 days, with the aim of reducing costs to people in the care of their ongoing chronic health conditions [5]. However, the full effect of prescription duration and dispensing quantity on the health care system, including costs and clinical outcomes, is debated [6]. The supply of larger quantities for subsidised medication, providing longer duration for each prescription, may be better for patient convenience and adherence, and lower costs for both patients and health care systems; while shorter durations may decrease medicines wastage and promote more frequent interactions with health care professionals. Dispensing quantity and prescription durations are commonly regulated by legislation, guidelines, and subsidy programmes in order to balance convenience, costs and safety [7–37]. This narrative review aims to summarise the current legislative and regulatory framework in Australia and abroad, as well as evidence on the potential impact that dispensed quantity and prescription durations may have on patient outcomes and costs.

## Methods

We searched the PubMed database for peer-reviewed articles since January 1st, 1990, up to September 1st, 2023, using the search terms ‘prescription duration’[title], ‘*duration prescriptions’[title], ‘prescription length’[title], ‘prescription period’[title], ‘extended prescription’[title], ‘repeat prescription’[title], ‘repeat prescribing’[title], and ‘dispensing duration’[title]. In addition, for a selection of countries, we also searched for and asked national health care authorities or local experts via e-mail regarding dispensing and prescription duration limitations based on legislation, health service guidelines, and subsidy programme regulations. To achieve a representative sample, we included the world’s 5 most populous countries, as well as the most populous country from each continent [38]. We also included at least 10 countries with comparable healthcare systems with subsidised access to medicines, as in Australia. Other countries were included based on accessibility of information online, responsiveness by national health care authorities, or connections with local experts.

**Fig. 1:**
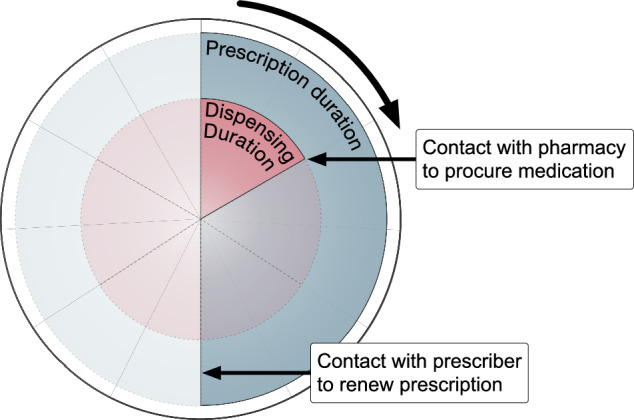
Schematic illustration of dispensing and prescription durations, exemplified with a prescription which includes two repeats. Prescription and dispensing durations may vary depending on country and medication. Figure only for illustrative purpose

In this paper we define ‘prescription duration’ as the entire period of treatment, based on the quantity of medication, for which a healthcare provider may prescribe a medication (which may or may not include several repeat dispensings), and ‘dispensing duration’ as the period of treatment for which a pharmacist may dispense the medication at a single time point (Fig. 1). Although these time periods are varyingly defined in days, weeks, months, or years, we will specify prescription durations in the approximate number of months, and dispensing durations in the approximate number of days, as these are the most used units of time in regulatory frameworks. Duration limitations of various countries and regions described in this paper are based on legislation, guidelines, subsidy programmes, and/or in some cases customs, and thus are not always comparable, but rather intended as an overview of the global heterogeneity. Finally, limitations on maximum dispensing or prescription durations may differ from the most used durations because of factors such as culture, customs, local clinical practice and financial or medication supply related constraints, which may result in shorter durations; and medicines package sizes, which may result in either shorter or longer durations.

## Results

**Fig. 2:**
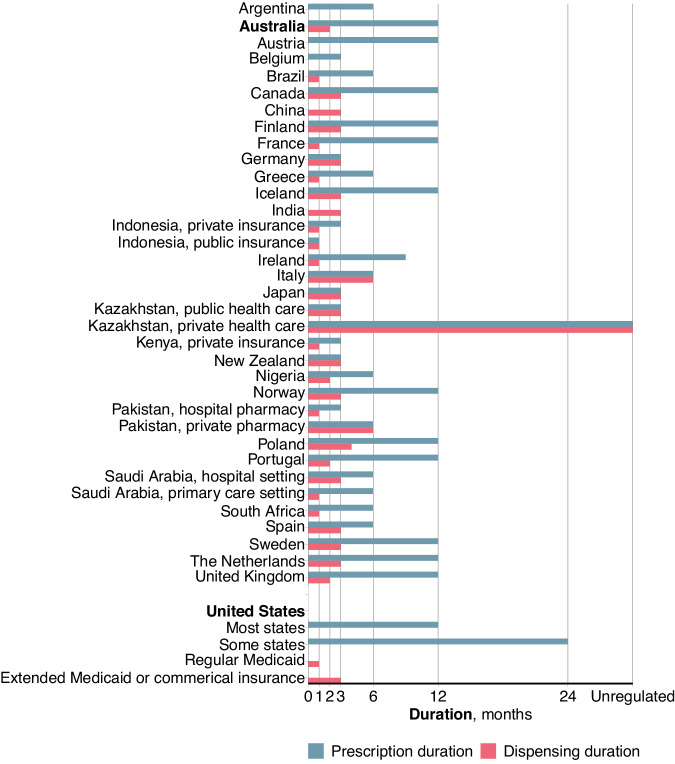
Maximum prescriptions and dispensing durations of medicines for chronic conditions, by country and setting. Duration limitations are by law, guidelines, or subsidy programme requirements, or if not regulated, as advocated or customary. For some countries, regional examples are used when regulations vary depending on region. Actual durations may be shorter or longer depending on practicing cultures and traditions, as well as medicines package sizes, adherence, and dosage

We retrieved information on dispensing and prescription duration limitations from 32 countries [39]. In most countries for which we were able to retrieve relevant information, dispensing durations were either 30, 60 or 90 days, and prescription durations were either 3, 6 or 12 months (Table 1 and Fig. 2) [7–37]. In Australia, the dispensing duration was recently extended from 30 to 60 days for a selection of medicines for chronic health conditions. The number of repeats was kept unchanged, so that the prescription duration for these select medications was extended from e.g., 6 to 12 months [5, 9]. Notably, Italy was the only country with a regulated dispensing duration of 180 days, which was twice that of any other country with regulated dispensing durations [29].

**Table 1:** Maximum dispensing and prescription durations of medicines for chronic conditions, by country and setting

Country	Regulation(s)
Argentina	In Argentina, prescription duration varies between 1 and 6 months depending on whether the individual is covered by social security or not, and whether the social security is state or pre-paid (Personal communication with Dr Walter Espeche, 2023-12-20).
Australia	Under the Pharmaceutical Benefits Scheme regulations (for eligible drugs for chronic conditions, such as hypertension), prescription duration is limited to 6 months, and dispensing duration is limited to 30 days [8]. Since September 1st 2023 some chronic disease medications (including those for hypertension) are exempt and can be prescribed with prescription durations of up to 12 months and dispensing durations of up to 60 days [8].
Belgium	Prescription durations are unlimited, but to be covered by public insurance, they are limited to 3 months [19].
Brazil	Within the public health care system, in which around 75% of the Brazilian population receives treatment, prescription duration is limited to 6 months, and dispensing duration is limited to 30 days [33].
Canada	Prescription durations vary depending on provinces, but in e.g. Ontario, a prescription is legally limited to a 12 months’ supply [10]. Regulations on dispensing durations also vary, but for chronic conditions with long-term prescriptions, the maximum dispensing duration is most often between 90 and 100 days if benefits are to be paid [9].
China	The maximum dispensing duration varies between provinces. For medication for chronic illnesses such as hypertension, however, it is often between 60 and 90 days (Personal communication with Professor Jiguang Wang, 2023-10-26).
Finland	Although prescriptions durations are limited to 24 months, with a maximum of 3 repeats [21], the state social security is only valid for dispensing durations limited to 3 months [20]. Thus, with 3 repeats, the maximum prescription duration is 12 months.
France	Prescription durations are limited to 12 months and dispensing durations are limited to 1 month [16]. Packages which correspond to longer treatment periods allow for dispensing durations of up to 3 months [22].
Germany	Both prescription duration and dispensing duration are limited to maximum 3 months for individuals covered by the public insurance (Personal communication with Dr Tina Stellwag, 2023-11-16).
Greece	Maximum dispensing duration is 30 days, with 5 repeats possible, so that the prescription duration is maximum 6 months (Personal communication with Professor George Stergiou, 2023-10-25).
Iceland	Prescription durations are limited to maximum 12 months, and dispensing durations to maximum 100 days [24].
India	Government has recently urged clinicians to prescribe medicines with dispensing durations of 3 months for chronic diseases. However, adequate supply of medicine is a prerequisite to implement this [25]. Furthermore, the private sector is a dominant player in healthcare provision in India, and prescribing practices vary considerably between private and public healthcare settings, as well as among states (Personal communication with Dr Mohammad Abdul Salam, 2023-11-29).
Indonesia	Both prescription and dispensing duration in primary care covered by public health insurance is usually limited to 1 month. For individuals with private insurance, prescription duration is often up to 3 months, limited by custom rather than legislation (Personal communication with Dr Anthony Sunjaya, 2023-10-19).
Ireland	Prescription durations were extended from 6 to 9 months during the COVID-19 pandemic, and these extensions are still valid but under review (E-mail communication with the Pharmaceutical Society of Ireland, 2023-09-15). Guidelines for general practitioners recommend a maximum prescription duration of 12 months [27]. Dispensing durations are limited to 30 days under the General medical services and Community drug schemes [26].
Italy	Prescription durations are limited to maximum 6 months, and all medications prescribed can be retrieved at once from the pharmacy, thus resulting in a dispensing duration of 180 days [28].
Japan	Prescription duration is not regulated, but is in clinical practice limited to 3 months. Dispensing duration is also limited to 3 months (Personal communication with Professor Satoshi Hoshide, 2023-11-22).
Kazakhstan	Medications for common chronic conditions are sold over the counter without the need of a prescription, and thus patients who choose to use the private healthcare services may procure whichever duration of treatment that they desire from the pharmacy following the doctor’s recommendations. For those using public health care, dispensing and prescription durations are usually limited to between 1 and 3 months (Personal communication with Dr Yelena Khegay, 2023-10-31).
Kenya	Prescription and dispensing durations are oftentimes limited by stock shortages rather than regulations, and often to as short as 14 days. With private insurance, prescription duration is usually limited to 3 months and dispensing to 30 days (Personal communication with Professor Elijah Ogola, 2023-10-26).
New Zealand	Prescription duration is limited to 3 months and dispensing duration is limited to 90 days [34].
Nigeria	Regulations vary between health centres, with prescription durations usually limited to 3-6 months, and dispensing durations to 1-2 months, if to be covered by insurance. However, most patients are limited by personal financial conditions to retrieve at most 1 months’ supply at once (Personal communication with Professor Mark Huffman and Professor Dike Ojji, 2023-09-21).
Norway	Prescription duration is limited to 12 months, and dispensing duration is limited to 90 days [35].
Pakistan	Hospital pharmacies generally limit prescription durations to 3 months and dispensing durations to 30 days. However, private pharmacies, which are more commonly used, do not enforce any general limitations, although both prescription and dispensing durations are sometimes limited to 6 months (Personal communication with Professor Bilal S. Mohydin, 2023-11-18).
Poland	Prescription duration is limited to maximum 12 months, and dispensing duration is limited to 120 days (E-mail communication with the Ministry of Health [Ministerstwo Zdrowia] of Poland, 2023-11-12). The dispensing duration was reduced as of the 1st of November 2023 from 365 to 120 days, although the subsequent period can be filled after three-fourths of the previous period (Personal communication with Professor Dagmara Hering, 2023-11-27).
Portugal	Prescription duration is limited to maximum 12 months, and dispensing duration is limited to 60 days (E-mail communication with the Ministry of Health [Gabinete do Ministro da Saúde] of Portugal, 2024-01-31).
Saudi Arabia	The maximum prescription duration commonly used is 6 months. The dispensing duration varies depending on care giver, but is commonly 90 days in hospital settings, with 1 repeat, and 28 days in primary care settings, with 5 repeats (Personal communication with Dr Ahmed Bahamdan, 2023-10-27).
South Africa	Prescription duration is limited to 6 months [15]. Dispensing duration is typically around 30 days [30].
Spain	Prescription duration for chronic conditions is limited to 6 months and dispensing duration is limited to 90 days [13].
Sweden	Prescription duration is limited to 12 months [11]. Dispensing duration is legally limited to 360 days, but for medications to be subsidised by the government, dispensing duration is limited to 90 days. Additional medications may be retrieved once at least two thirds of the previous period has passed [12].
The Netherlands	Prescription duration is limited to 12 months and dispensing duration to 90 days (Personal communication with Dr Joost Moerman, 2023-10-27).
United Kingdom	For electronic prescriptions, prescription duration is limited to 12 months, and dispensing duration is usually limited to 56 days [14].
United States	Neither the prescription nor dispensing duration is limited by federal law [36]. In some US states, prescription duration is limited to 24 months [6]. Dispensing durations through Medicaid was originally limited to 30 days, but extended to 60 or 90 days in some states during the COVID-19 pandemic, although sometimes limited to a list of maintenance drugs such as blood pressure lowering medications [31]. However, since the pandemic abated, at least some states have reverted these extensions and returned to more restrictive dispensing durations [32]. A majority of commercial insurances offers dispensing durations of up to 90 days [31]. State of Texas: There are no specific rules which specifically address prescription durations for dangerous drugs. However, insurance companies will most often only accept payment for dispensing durations limited to 1 month, and prescription durations limited to 1 year (E-mail communication with the Texas State Board of Pharmacy, USA, 2023-10-05). State of New York: Under Medicaid, for most maintenance medications, prescription duration is limited to 12 months and dispensing duration is limited to 90 days (E-mail communication with the Medicaid Pharmacy Policy Division of Program Development and Management, Office of Health Insurance Programmes, New York State Department of Health, USA, 2023-11-18).

### Medication package sizes

In some countries, regulations also include exemptions for medications that are only available in larger packages. In Sweden, longer than 90-day dispensing durations are allowed within the scope of the drug benefits programme, if package sizes cover more than 90 days’ supply [13]. Likewise in Spain, dispensing of electronic prescriptions may cover at most 30 days, unless the package size corresponds to a longer period [14]. For the same reason, some medications are dispensed for longer durations than what is generally stipulated also in Australia, e.g., indapamide which is only sold in packages of 90 tablets [40]. Another example of regulatory adjustments relating to medicines package sizes is in Ireland, where an additional dispensing is allowed once a year when packages only contain 28 tablets, so that the full year can be covered by 12 dispensings [41]. It has also been discussed whether changes in the size of medication packages may influence dispensing duration patterns [42, 43]. However, it could also be the case that the size of medication packages is adapted by producers to regulations in the market in which they are sold. For example, the blood pressure lowering medication, losartan, is available in packages that cover treatment durations of between 28 and 100 days in Sweden, but only 30-day packages are available in Australia [44–46].

### Effects on costs and use of resources

When comparing 30 vs 60 or 90-day dispensing durations, studies show that longer durations lead to increased medicine waste, but this seems to be to a small degree [39, 42, 47, 48]. The evidence of increased medicines waste is considered as weak, and none of the available studies were randomised controlled trials [39, 47]. Reasons for medication returns (for disposal) include expired medications, change of drug or dose (strength), discontinuation of medication, side effects or lack of treatment effect, and return of medicines after the death of a patient [49]. Policy change in the UK based on studies on medicines waste has later been criticised for not taking into account other costs and behavioural effects of shorter dispensing durations, nor the increased use of generic branded medicines which has substantially decreased medicine costs and thus the financial implications of medicines waste [48]. Australia, like many countries, has a return of unwanted medicines programme, but most individuals dispose of unused medications in the household rubbish or sewerage [50]. Also, most returned medicines are for acute rather than chronic conditions [50].

Shorter dispensing durations entail higher costs for pharmacy services such as handling fees, which offset the savings of less medicines waste [42, 47, 51]. This is true in both the UK and the US, despite large differences in pharmacy fees [47]. In fact, with short dispensing durations, pharmacy charges in some circumstances may exceed the cost of the actual treatment [48]. For example in Australia in 2022, for hypertension alone, electronic prescription fees and dispensing fees incurred A$ 238 million and 370 million, respectively, which was more than half of the total spending on hypertension treatment [4]. In Australia, ‘dispensing fee’ concerns the direct costs of dispensing (handing over to patient, instructing patient, charging patient), and the ‘Administration, Handling and Infrastructure fee’ concerns the indirect costs of dispensing (administration, infrastructure and handling). Shorter dispensing durations may also increase direct costs for the patients, including costs of transportation to and from the pharmacy, as well as the time consumed [52]. Whether frequent prescription fills also lead to loss of productivity or working hours has not been studied. The need to frequently visit the pharmacy and general practice may be particularly challenging for patients with limited access to health care, such as those living in rural and remote areas [47, 53]. This aspect has particular relevance for Australia, where around 7 million people (or 28% of the population) live in rural and remote areas, including many diverse locations and communities [54]. While some countries like Australia have location rules that support pharmacies being geographically distributed according to the population, access to general practitioners can be limited by supply and waiting times [55–57].

In relation to the extension of dispensing durations in Australia, pharmacists have raised concerns that longer durations could strain their financial margins, and that this could result in fewer pharmacies and more restricted opening hours [58]. In the UK, reimbursement schemes for pharmacies could be a source of similar opposition [59]. Such concerns are important to address. Pharmacists have a key role to play as part of team-based care. Their interaction with patients can reduce medication-related harm, help promote uptake of generic branded medicines (to save patient out-of-pocket payments as well as government costs for subsidy programmes), and advise patients on proper medication use to improve adherence, which may both save costs and improve health outcomes [60, 61]. For example, in a cluster randomised trial in Sydney, Australia, pharmacists supporting asthma patients’ in using correct inhalation techniques improved asthma control [62]. However, whether dispensing durations would affect the benefits of such interventions has not been studied.

Finally, a shorter prescription duration may increase the workload and time-burden on busy general practitioners, and patients themselves may experience the process of requesting a renewed prescription as a waste of valuable time for the physician despite the value of regular medical reviews [39, 47, 53]. In low- and middle-income countries with a poor physician-to-patient ratio, longer prescription durations could also free up limited healthcare resources so that the public health care system could increase its reach to a larger proportion of the population [26].

### Effects on medication supplies

Pharmacists in Australia have raised concerns that increased dispensing durations may disrupt medications supplies due to medicines shortages [63]. Medication supply issues do occur, as was particularly evident during the COVID-19 pandemic, but as is also on-going with the Australian medicine shortage reports database currently listing 44 medications as critical [64, 65]. However, current regulations in Australia already limit prescription and dispensing of medications with supply issues, and an increased dispensing duration does therefore not apply to such medications [64]. Furthermore, pharmaceutical companies that supply medicines in Australia under its subsidised access scheme are required to maintain a minimum stock holding [66]. Guidelines in some countries indicate that increased patient medication supplies may in fact counteract the negative impact of shortages. In Sweden, the recurring shortages of medications recently motivated the National Board of Health and Welfare to issue a recommendation that patients at all times keep an individual stockpile of at least 1 months’ medication supply [67]. This was proposed as a balancing between preparedness, patient safety, and the risk of medicines waste, and a way to reduce the burden on both the health care and pharmacy systems, including to reduce the risk of overload if a serious event would occur, whether in time of peace or war [67, 68]. Thus, to increase the resilience of the health care system, longer dispensing and prescription durations may be beneficial, whilst in the event of an acute shortage, a temporary restriction on dispensing durations may be necessary.

### Effects on adherence and health outcomes

Observational studies suggest that longer dispensing durations are associated with better medication adherence, where most studies have specifically looked at medications of chronic conditions such as hypertension, hyperlipidaemia, and diabetes mellitus [39, 42, 47]. However, evidence is limited by difficulties in separating days of supply from the measures of adherence used, and none of the studies were randomised controlled trials. In one study including over 200,000 newly diagnosed patients with hypertension, comparing prescription durations of less than 28 days with 29 days or more, longer duration prescriptions were associated with better long-term adherence measured by the proportion of days covered [69]. The relatively short prescription durations compared in this study were due to the study of treatment initiation, rather than a stable repeat treatment. More recently, a single centre pre-post implementation study in Thailand of patients undergoing treatment with statins or diabetes medication found that a change from 30- to 90-day prescription periods (as part of universal health coverage) increased adherence (assessed by the medication possession ratio), and to a level on par with similar people at the same hospital who’s health insurance consistently provided 90-day prescriptions [70].

Improved medication adherence is positively associated with health outcomes, including reduced all-cause mortality, as well as reduced hospitalisation and thus health-care costs [39, 47, 71]. However, although one study reported improved serum cholesterol concentrations with 60- vs 30-day dispensing durations, the direct association between prescription time and clinical outcomes has not been sufficiently studied [39, 47].

Finally, the upper limit of prescription durations is sometimes explicitly motivated in regulations by how often it is considered necessary for a clinician to follow-up on a chronic condition, thus intended to work both as a limit of drug retrieval for patients and as a reminder to book a follow-up for clinicians [72].

### Patient experiences

Patients describe repeat prescriptions as time-consuming and life-disrupting, and that the effort needed to fill them is increased with 30-day vs longer dispensing durations [59]. In a study of patients with thyroid disease in the UK, 59% of respondents expressed dissatisfaction with 28-day dispensing durations, mainly because of inconvenience and interference with work, but also because it reminded them of their chronic health condition [53].

## Discussion

Prescription and dispensing durations vary significantly between countries. The current standard dispensing quantity supplied which provides a duration of treatment of 30 days, and for some medicines 60 days, is not unique to Australia, although many countries allow dispensing durations to 90 days (Fig. 2 and Table 1). Evidence on the impact of dispensing and prescription durations is limited in quality, but suggests that longer dispensing durations are less costly to the patient (and funder) and improve adherence compared with shorter dispensing durations [39, 42, 47, 48, 51, 52, 69, 70] (Table 2). Selective use of shorter dispensing durations for medications that are particularly costly, have a higher environmental burden (e.g., antibiotics), are subject to medicines shortages, or have a risk of dependence, is reasonable and can be regulated but also ultimately decided upon at the discretion of the prescribing healthcare provider. However, for chronic health conditions such as a stable patient with hypertension or hypercholesterolaemia, in particular when an effective treatment response has been ensured, the option of longer dispensing durations could increase adherence and decrease costs for both the patient and the entire health care system. However, high-quality studies, as well as thorough evaluation of the current transition from 30 to 60 day dispensing durations in Australia, are needed to confirm whether these suggestions hold true.

**Table 2:** Summary of studies on the effects of prescription and dispensing durations

Outcome measure	Country	Strength of evidence
**Benefits of longer dispensing duration**		
Increased medication adherence	China, Thailand, United Kingdom, USA	Weak. None of the available studies were randomised controlled trials, and had methodological limitations in measuring supply and adherence [39, 42, 47, 69, 70].
Reduced costs for pharmacy services	United Kingdom, USA	Weak. Based on cost estimates and retrospective cost analyses, but not directly measured in any study [42, 47, 48, 51].
Reduced use of health care resources, including physicians’ time	India, United Kingdom, USA	Weak. Based on patient experience and cost estimates, but not directly measured in any study [26, 39, 47, 53].
Reduced inconvenience for patients	United Kingdom	Weak. Based on patient experience, but not directly measured in any study [53, 59].
**Benefits of shorter dispensing duration**		
Reduced medicine waste	United Kingdom, USA	Weak. None of the available studies were randomised controlled trials [39, 42, 47, 48].
**Unknown effects (not studied at all, or insufficiently studied)**		
Loss of productivity, working hours and other direct costs for patients		Patients experience shorter prescription durations as life disruptive, including multiple journeys to the pharmacy [53, 59], but we could not find any study in which the effects on productivity and working hours were directly measured.
Change in frequency or quality of pharmacy supported interventions		The benefit of pharmacy support (e.g., encouraging medication adherence) has been studied in several countries e.g., Australia, Brazil, Denmark, Germany, Ireland, The Netherlands, Poland, Portugal, Singapore, Sweden, Switzerland, the United Arab Emirates, the United Kingdom, and the USA [60–62]. However, we could not find any relevant studies on the relation between prescription durations and pharmacy support.
Change in frequency or quality of medical reviews by physicians		No relevant studies were found.
Effects on medicines supplies and potential shortages		No relevant studies were found.
Effects on health outcomes		Increased adherence is known to benefit health outcomes, but we could not find any relevant studies on the direct association between dispensing durations and health outcomes.

Community pharmacies have raised concern regarding the financial effects on them of extended dispensing durations. Pharmacists provide valuable services, including dispensing of drugs, but also clinical services such as medication reviews and guidance to patients on adherence and proper medication use. Although this is equally important regardless of dispensing durations, adequate compensation needs to be ensured for this service to be sustained.

Finally, to understand the full effects of different prescription and dispensing durations on medication adherence, medicines supplies, health outcomes, and costs for both the patient and the health care services, and to improve the quality of evidence of what previous studies have thus far suggested, randomised controlled trials are needed [47]. Standardised measurements of medication adherence and waste, as well as comparisons of several different dispensing durations, has also been requested [47]. In fact, the recent 2023 Global Report on Hypertension from the World Health Organization suggests 90- or 180-day dispensing durations for patients with hypertension who are stable [73]. Thus, it would be important to compare the most common dispensing durations such as 60 days and 90 days, which are used in 21 of the countries we have surveyed, but also extended durations such as 120 or 180 days.
